# Nonlinear estimation of BOLD signals with the aid of cerebral blood volume imaging

**DOI:** 10.1186/s12938-016-0137-6

**Published:** 2016-02-20

**Authors:** Yan Zhang, Zuli Wang, Zhongzhou Cai, Qiang Lin, Zhenghui Hu

**Affiliations:** College of Optical and Electronic Technology, China Jiliang University, Xueyuan Street 258, Hangzhou, 310018 China; College of Optical Science and Engineering, Zhejiang University, Zheda Road 38, Hangzhou, 310027 China; Center for Optics and Optoelectronics Research, College of Science, Zhejiang University of Technology, Liuhe Road 288, Hangzhou, 310023 China

**Keywords:** Blood volume fraction, Cerebral blood volume imaging, Dynamic causal modeling

## Abstract

**Background:**

The hemodynamic balloon model describes the change in coupling from underlying neural activity to observed blood oxygen level dependent (BOLD) response. It plays an increasing important role in brain research using magnetic resonance imaging (MRI) techniques. However, changes in the BOLD signal are sensitive to the resting blood volume fraction (i.e., $V_0$) associated with the regional vasculature. In previous studies the value was arbitrarily set to a physiologically plausible value to circumvent the ill-posedness of the inverse problem. These approaches fail to explore actual $V_0$ value and could yield inaccurate model estimation.

**Methods:**

The present study represents the first empiric attempt to derive the actual $V_0$ from data obtained using cerebral blood volume imaging, with the aim of augmenting the existing estimation schemes. Bimanual finger tapping experiments were performed to determine how $V_0$ influences the model estimation of BOLD signals within a single-region and multiple-regions (i.e., dynamic causal modeling). In order to show the significance of applying the true $V_0$, we have presented the different results obtained when using the real $V_0$ and assumed $V_0$ in terms of single-region model estimation and dynamic causal modeling.

**Results:**

The results show that $V_0$ significantly influences the estimation results within a single-region and multiple-regions. Using the actual $V_0$ might yield more realistic and physiologically meaningful model estimation results.

**Conclusion:**

Incorporating regional venous information in the analysis of the hemodynamic model can provide more reliable and accurate parameter estimations and model predictions, and improve the inference about brain connectivity based on fMRI data.

## Background

Functional magnetic resonance imaging (fMRI) offers a noninvasive technology to examine hemodynamic signals in the cerebrovascular system. The hemodynamic balloon model was introduced in 1998 to reveal the coupling dynamics between neural activity and blood oxygen level dependent (BOLD) responses by Buxton et al. [1]. The balloon model describes the causal mechanisms within a hemodynamic process in a certain region of interest (ROI) during brain activation. BOLD responses can be observed via the dynamic changes in cerebral blood volume (CBV) *v*, cerebral blood flow *f*, and vein deoxyhemoglobin (dHb) content *q*. This model is especially helpful in understand the potential consequences of interactions between physiological mechanisms. Since the inception of this model, there has been growing interest in using it to interpret observed fMRI data. The model can be used to infer biologically meaningful parameters that could be employed to investigate the changes in underlying physiological variables during brain activation [2–5], restrict the activation detection process with classic statistical techniques [6, 7], and deduce similar systems or different driving conditions [8–11].

The primary causes of unreliability in model estimation is that the BOLD fMRI technique is sensitive to changes in the signal from venous blood. The change in the signal intensity of a particular voxel is strongly dependent on what fraction of the voxel the vessel occupies. Moreove, changes in BOLD signal intensities during task activation are related not only to multiple physiological states but also regional vessel occupancy, including capillaries and large veins. Indeed, the evaluation of model structure also indicates that the blood volume fraction (BVF) greatly influences the uncertainty of model output [12]. However, this problem has been ignored in all previous studies. Most studies performed to data have avoided the ill-conditioning problem simply by employing a physiological plausible value of $V_0=0.02$ instead of investigating the actual value in a particular ROI [2–5, 7, 13, 14] or throughout the brain [6, 15].

Given the importance of the true BVF, efforts are needed to incorporate actual vascular information of the voxel in the hemodynamic model estimation. Firstly, when a voxel includes only brain tissue, the assumption of $V_0=0.02$ is reasonable [2, 16]. However, when a voxel is mostly or totally occupied by a vessel or vessels, the value might typically be above 0.6 [17]. Secondly, voxels associated with a larger amount of blood are always more likely to show significant BOLD activation due to the inherent nature of the fMRI technique. In this situation, employing an unrealistic $V_0$ value might yield an unreliable model that does not reflect the physiological reality. This illustrates the importance of taking into account the actual BVF during the estimation procedure.

Several methods have been applied in attempts to obtain the true BVF. We recently showed that magnetic resonance angiography (MRA) might provide a method for roughly estimating the BVF value [18]. The results inferred that the $V_0$ value in a voxel consists of two derivative components: (1) a constant tissue blood volume component $V_s=0.02$, which is the small-vessel blood volume that includes capillaries and small postscapulaes, and (2) a variable large blood vessels component $V_l$, which is the blood volume of large blood vessels. However, this method has not been used to obtain the actual $V_0$ directly. Indeed, the regional CBV can be measured by another imaging modality, called the dynamic susceptibility contrast (DSC) material-enhanced gradient-echo (GE) MR technique [19]. The present study therefore augmented the true BVF acquired from CBV imaging in order to focuses on the influence of $V_0$ on hemodynamic model estimation and the importance of using the true BVF in the analysis.

This paper is organized as follows. Firstly, we briefly review the hemodynamic Balloon model which constitutes the fundamental component of hemodynamic model estimation. Secondly, we explain the important influence of $V_0$ with the adoption of a realistic value obtained from the CBV imaging technique. Lastly, the influence of $V_0$ on model estimation within a single-region and multiple-regions according to the results of a classic bimanual finger tapping experiment is discussed in terms of the impacts of the actual $V_0$ on parameter estimates and state-space reconstruction.

## Hemodynamic balloon model

The hemodynamic balloon model describes the dynamic interrelationship between the blood flow *f* (neural activity to changes in flow), the regional blood volume information *v* (changes in flow to changes in blood volume and venous outflow), and the vein dHb content *q* (changes in flow, volume and oxygen extraction fraction to changes in dHb). The hemodynamic process can be described as the follows:

1 $$\begin{aligned} {\left\{ \begin{array}{ll} \ddot{f}=\epsilon u(t)-\frac{\dot{f}}{\tau _s}-\frac{f-1}{\tau _f} \\ \dot{v}=\frac{1}{\tau _0}(f-v^{1/\alpha }) \\ \dot{q}=\frac{1}{\tau _0}\left( f\frac{1-{(1-E_0)^{1/f}}}{E_0} -v^{1/{\alpha }}\frac{q}{v}\right) , \end{array}\right. } \end{aligned}$$

where $\tau _s$ reflects signal decay, $\tau _f$ is the feedback autoregulation time constant, $\tau _0$ is the transit time, $\alpha$ is a stiffness parameter, $\epsilon$ is the neuronal efficacy, *u*(*t*) is the neuronal input, and $E_0$ represents the resting oxygen extraction fraction. The variables *f*, *v*, and *q* are expressed in normalized form, relative to resting values. The balloon model can account for the hemodynamic responses in sparse, noisy fMRI measurements [12, 15]. However, since the above describing equations contain a second-order time derivative, we can introduce a new variable $s=\dot{f}$ to express this hemodynamic system as a set of four first-order ordinary differential equations. Then the observed response BOLD signal could be expressed as follows:

2 $$\begin{aligned} {\left\{ \begin{array}{ll} y(t)=V_0(k_1(1-q)+k_2(1-\frac{q}{v})+k_3(1-v)), \\ k_1=7E_0, \quad k_2=2, \quad k_3=2E_0-0.2. \end{array}\right. } \end{aligned}$$

**Fig. 1:**
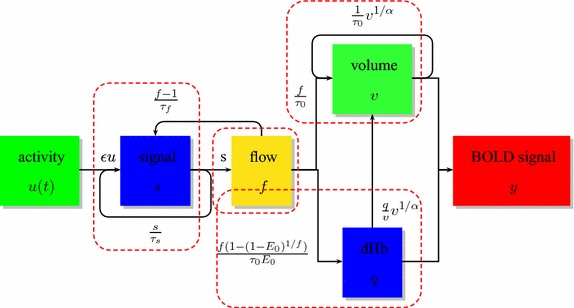
Schematic illustration of the hemodynamic balloon model. This model consists of three linked subsystems: (1) neural activity *u*(*t*) to changes in the cerebral blood flow *f*, the second-order time derivative equation is written as a set of two first-order time derivative equations by introducing a new state variable $s=\dot{f}$; (2) changes in flow *f* to changes in the cerebral blood volume *v*; (3) changes in flow *f*, volume *v* and oxygen extraction fraction to changes in the veins in the vein dHb content *q*

This equation is appropriate when using an fMRI machine 1.5-T magnet. The observed *y* is normalized relative to the value at rest, and $V_0$ is the resting BVF [2]. Equations 1 and 2 consist of the architecture of hemodynamic input-and-output system. The model architecture is depicted in Fig. 1.

**Fig. 2:**
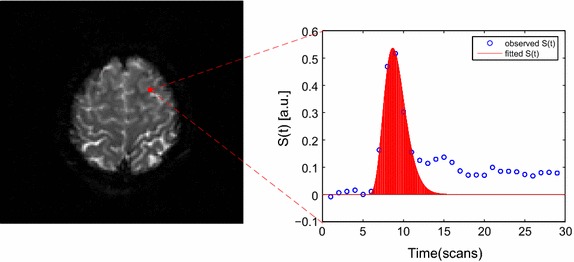
Example of an axial CBV image (*left*) and the observed signal-intensity-versus-time curves (*S*(*t*), *blue circles in right graphic*) and fitted concentration–time curves (*red line in right graphic*). *Red area* denotes the estimated $V_0$

The BOLD response is associated with all of these parameters, but, we know that parameter $V_0$ can not be identified along with other parameters simultaneously, instead only their product. Most previous efforts have imposed a physiologically plausible value of $V_0= 0.02$ to handle the ill-conditioned nature of the problem [2–10]. Changes in the BOLD signal are strongly affected by $V_0$, and so an unrealistic $V_0$ may lead to unreliable model parameter estimation.

## Experiment

Two human subjects participated in this study. The experiment was approved by the Health Sciences Research Ethics Committee of Zhejiang University, and written informed consent was obtained from both subjects. Functional images were acquired on a 1.5-T scanner using a standard fMRI echo planar imaging protocol (resolution: $64\times 64$ matrix; repetition time $\mathrm{TR}=2$ s). In total, 110 acquisitions were made in a block-designed finger tapping experiment, giving 11 20-s blocks. The conditions for successive blocks alternated between rest and task performance, starting with rest. Furthermore, the CBV imaging sequence consisted of 30 T$2^*$-weighted images that were collected with a GE sequence (resolution: $128\times 128$ matrix; 0.1 mmol/kg Gd-DTPA administered using a powered injector). In order to achieve a sufficient signal-to-noise ratio and complete coverage of the brain, TR was increased to 3.1 s, since a typical value is 1 s. The other sequence parameters remained unchanged.

All CBV images were down-sampled to make their spatial resolution identical to that of the fMRI image, and thereby allow voxel-by-voxel curve analysis. Concentration–time curves were created for each voxel [20–23]. The calculated $V_0$ was then used in an existing data estimation procedure [24]. Figure 2 shows an example of an axial CBV image and the observed *S* and fitted concentration–time curves from one voxel. Data preprocessing and statistical analysis were performed using the SPM5 program (Wellcome Department of Cognitive Neurology, http://www.fil.ion.ucl.ac.uk/spm). The activation map was obtained by applying *t*-tests between all bimanual motor conditions and resting baselines with a cutoff for statistical significance of $P < 0.001$.

**Fig. 3:**
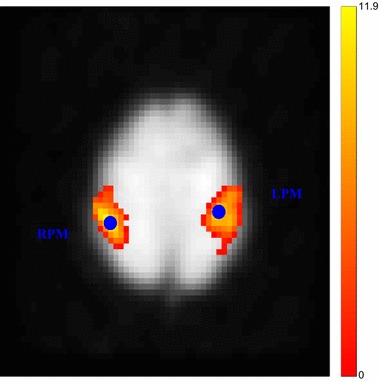
Selected ROIs based on typical activated areas detected in the bimanual tapping task. The activation map was obtained by applying *t*-tests between all bimanual motor conditions and resting baselines, with a cutoff for statistical significance of $P<0.001$

**Fig. 4:**
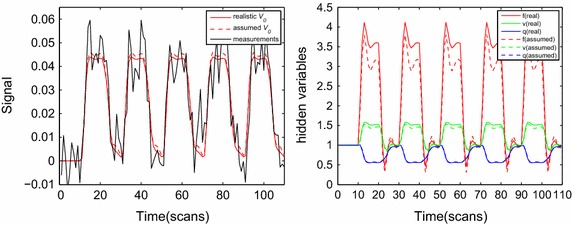
Estimated BOLD signal (**a**) and reconstructed physiological states (**b**) for the maximally activated voxel of subject 1. For comparison, model estimation was also performed with the typically assumed value of $V_0=0.02$. The real $V_0$ value of this voxel was 0.0172. It is evident that differences in the estimated physiological states are relevant to deviations from the actual BVF value

## Results

### Impact of BVF on single-region model estimation

We now compare and evaluate the respective impact of the realistic and assumed BVFs on hemodynamic model estimation within a single-region. Firstly, we chose the maximally activated voxel in the left primary motor cortex (LPM) on the basis of the analyzed fMRI data from SPM5 as the ROI (Fig. 2) and then defined the cluster based on faces and edges excluding corners in order for this voxel to have six neighbors. We extracted the ultimate time series to be analyzed by averaging over the time series of seven voxels. This procedure allowed the model parameters and state-space functions for each of the two subjects to be estimated. Furthermore, for the sake of simplicity, we assumed that the neural parameter had the same value throughout all trails: $\epsilon _1=\epsilon _2=\cdots =\epsilon _n$, where *n* denotes the number of trials (i.e., $n=5$ here). A control random search algorithm was applied in the parameter estimation procedure [25].

**Fig. 5:**
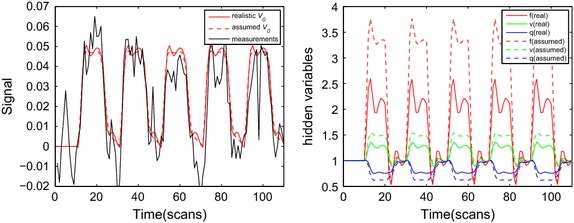
Estimated BOLD signal (**a**) and reconstructed physiological states (**b**) for the maximally activated voxel of subject 2. For comparison, model estimation was also performed with the typically assumed value of $V_0=0.02$. The real $V_0$ value of this voxel was 0.0308. It is also evident from this subject that differences in the estimated physiological states are relevant to deviations from the actual BVF value

**Fig. 6:**
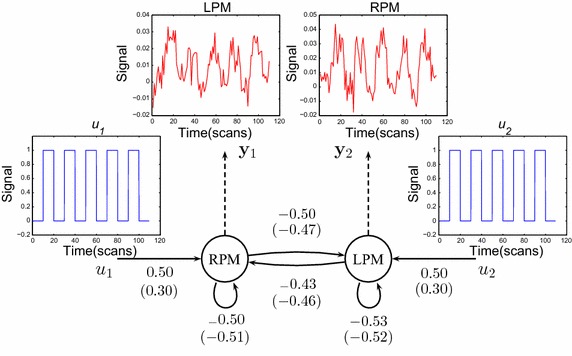
Results of a DCM analysis applied to the finger tapping experiment. The value indicates the connection strength ($a_{ij}$ in Eq. 3) in DCM. The coupling parameters calculated with the real $V_0$ are shown alongside the corresponding connections. The values in brackets indicate the deviations from parameters estimated when using the assumed $V_0$. $V_0=0.0185$ in the LPM, $V_0=0.0308$ in the RPM, and the assumed $V_0=0.02$ in both areas. $u_1$ and $u_2$ represent external inputs to the system, $y_1$ and $y_2$ are the hemodynamic observations, and *arrows* indicate connections

Figures 4 and 5 show the BOLD signal and underlying physiological variables of the two subjects for the real $V_0$ derived from CBV imaging in the maximally activated voxel. The estimated BOLD signal and state variables for an assumed value of $V_0=0.02$ are also drawn in Figs. 3 and 4 (as dashed lines). The comparison indicates that the assumed and true $V_0$ could produce similar BOLD estimates in terms of magnitude and shape, with only a slight distinction in the plateau period. This result is consistent with those of previous studies involving the balloon model. However, we also found a large difference between the assumed and actual $V_0$ values in terms of the reconstructed physiological states. We can conclude that the intensity of changes in the underlying state variables with the assumed $V_0$ were double those with the true $V_0$; that is, underestimating $V_0$ produced an overestimation of the physiological state variables. Moreover, Figs. 4 and 5 indicate that a larger difference between actual $V_0$ and hypothetical $V_0$, resulted in a greater difference between estimated physiological state. This means that attention should be paid to ensuring that a realistic $V_0$ is used in model estimation. The presence of a larger amount of blood in an activated voxel magnifies the effects induced by neuronal activity, lead to an excessive signal for that voxel and unrealistic activity predictions. Similar BOLD changes in a voxel associated with larger veins will change *f*, *v*, and *q* less than for a voxel with a smaller blood fraction. Most activation detection techniques are only capable of indicating the neural activity from changes in BOLD signal or activity maps, and they do not direct infer whether the underlying physiological variation is closely related to $V_0$ and actually reflects neural activity. Under this circumstance, the use of an arbitrary value of $V_0$ will influence the spatial specificity of fMRI signals in statistical testing. However, we can assume that functional activated regions induced by an experimental event rather than large regional amounts of blood and the employment of an unrealistic $V_0$ are suitable when fMRI signal estimation and activation detection are exclusively needed.

Table 1 indicates that the uncertainty of $V_0$ induces changes in other parameters, with $V_0$ exerting a complicated, nonlinear, and inconsistent influence on the entire hemodynamic process. Table 1 also indicates that $V_0$ has a greater influence on the estimated neuronal efficacy parameter $\epsilon$ than on the other parameters ($\epsilon$ is 0.3910 with the true $V_0$, and 0.9089 with the hypothetical $V_0$). A previous study found that the uncertainty of model output was more sensitive to variation of $\epsilon$ than those of other parameters, except $V_0$ [12]. The defined $\epsilon$ represents the efficacy with which neural activity causes an increased BOLD signal. As a consequence, if we could use the true $V_0$, the estimated $\epsilon$ could offer a better and more intuitive reflection of the activation level, enhancing the functional specificity of fMRI.

**Table 1:** Model parameters estimated using the true value ($V_t$) and a typical assumed value ($V_a$) for the maximally activated voxels of two subjects

Subject	Maximally activated voxel	Model parameters				
		$\epsilon$	$\tau _s$	$\tau _f$	$\tau _0$	$E_0$
1	$V_t=0.0172$	0.8858	1.9067	2.9133	4.7506	0.5579
	$V_{\alpha }=0.02$	0.6598	2.6444	3.1977	5.2499	0.4388
2	$V_t=0.0308$	0.3910	3.3874	2.8647	4.5286	0.6288
	$V_{\alpha }=0.02$	0.9089	1.6889	2.5726	4.4636	0.6569

### Impact of BVF on dynamic causal models

As for balloon model research, dynamic causal modeling (DCM) has been introduced to explore effective connectivity based on hemodynamic observations [8, 9]. DCM extends the balloon model from a single region to multiple regions by utilizing a multiple-input, multiple-output system. Single-region model estimation supposes that the extrinsic experimental input consistently accesses all brain regions and that a certain brain area only receives input in this way ($\epsilon u$ in Eq. 1), whereas DCM assumes that responses ($x_i$ in Eq. 3) are elicited by two distinct inputs sources: the extrinsic influence of the sensory input ($\epsilon u$ in Eq. 3) and the intrinsic influence of the interaction regions ($a_{ij} x_k$ in Eq. 3). In other words, DCM uses estimated neural activities (internal and external) to evaluate the causal correlation among brain areas. While the uncertain $V_0$ has an important influence on parameter $\epsilon$ in the hemodynamic model, it is interesting to know how the $V_0$ influences DCM. In this study we therefore also investigated the effect of $V_0$ on DCM.

We constructed the simplest two-region hierarchical system in order to demonstrate the significant effect of BVF on the DCM system. From the two brain areas that interact with and influence each other, we could measure the observed BOLD signals that each of the two regions produced, the relationship can be expressed as follows:

3 $$\begin{aligned} {\left\{ \begin{array}{ll} \dot{x}_1=a_{11}x_1+a_{12}x_2+c_{11}u_1 \\ \dot{x}_2=a_{22}x_2+a_{21}x_1+c_{22}u_2\\ \end{array}\right. } \end{aligned}$$

where $x_1$ and $x_2$ are the neuronal dynamics in two regions, $u_1$ and $u_2$ represent external inputs to the system, $a_{11}$ and $a_{22}$ represent the internal connectivity within a region without input, $a_{12}$ and $a_{21}$ encode the fixed inter-region connectivity without input, and $c_{11}$ and $c_{22}$ embody the extrinsic influences of input on neuronal activity. One can augmented the state vector consisting of the model parameters at two regions by concatenating them into a single higher dimensional state space and the measurement vector was also expanded to include two observations in two areas [8]. In the experiment, we adopted a 0–1 square-wave function as two inputs, and the system output was two time series from two regions, $x_1$ and $x_2$. While attempting to determine the dimension of the parameters, a more efficient filtering strategy was used to deal with the model estimation problem [26, 27]. The estimation scheme employed for DCM is formally identical to that reported previously [5, 15]. The results of this analysis are presented in Fig. 5, in which the effective connections are presented as directed black arrows along with coupling parameters calculated with the real $V_0$ and assumed $V_0$. In order to construct the model system, we chose two regions in the left primary (LPM) and the right primary motor cortex (RPM) containing the two maxima of the activation map. The output region-specific time series comprised all adjacent (based on faces and edges but not corners) voxels of each maximum (a total of seven voxels), the location is shown in Fig. 2. The conflicts between the motor preparation were interpreted as inhibitory connections between the LPM and RPM [28, 29]. The fixed connectivity from the RPM to the LPM is actually slightly weaker than that from the LPM to the RPM. This indicates that backward influences (RPM to LPM) are stronger than forward connections (LPM to RPM). Furthermore, the fixed connectivity in the RPM is stronger than that in the LPM, indicating that the right path-way is used more frequently than the pathway on the left side. From Fig.6 we conclude that the two different $V_0$ have different impacts, with the largest deviation being about $40\,\%$ for the strength of the visual input to the LPM or RPM.

## Discussions

This study focused on the important but long ignored issue of how the resting cerebral BVF (i.e., $V_0$) impacts hemodynamic models. Previous studies have used a physiologically plausible value of $V_0= 0.02$ instead of exploring the actual $V_0$ in the model estimation procedure. However, the intensity of any hemodynamic signal change is greatly affected by the regional BVF, since the active domains subject to model estimation often overlap with those areas characterized by a large BVF [30]. Under such circumstances, an inaccurate $V_0$ may give rise to inaccurate estimates of the parameters and the reconstructed physiological state. This study used CBV imaging to augment the true $V_0$ calculated in the hemodynamic model. In order to show the significance of applying the true $V_0$, we have presented the different results obtained when using the real $V_0$ and assumed $V_0$ in terms of single-region model estimation and DCM. It was found that using the actual $V_0$, yielded more realistic and physiologically meaningful model estimation results.

The results obtained in this study indicate that $V_0$ has a rather complicated impact on estimated model parameters. Despite the BOLD responses being similar when using the assumed and real $V_0$, there was a huge difference in the estimated parameters and the derived physiological state in the ROI. Because the balloon model describes the causal mechanism of a hemodynamic system, its order is higher than the externally observable system, which results in poorly identifiable model parameters due to the nature of nonlinear optimization and temporally sparse sampling. These model parameters have clear physiological meanings, and they should be justified and interpreted with caution [13, 31]. If the actual $V_0$ is adopted, $\epsilon$ can be more reliably observe via fMRI measurements. Therefore, $V_0$ significantly influences the evaluations of brain connectivity. There have recently been extensive discussions on DCM and Granger causal modeling (GCM), with an emphasis on the connectivity among distributed brain systems [32–34]. In order to obtain a more robust understanding of brain causality, we used a biophysical model to eliminate signal bias in imaging procedure and variations of the hemodynamic response in diverse brain domains. However, an unrealistic $V_0$ might degraded such efforts.

A potential limitation of the present study is to the extent that $V_0$ as measured by CBV imaging is affected by the amount of blood associated with BOLD signals. We consider that both CBV imaging and the BOLD contrast have tiny difference in terms of the $V_0$. The former contains the volume of blood across arteries, capillaries, and veins, whereas the latter is relevant to capillaries and veins [35]. Although the arterial fraction of CBV is much less than the venous BVF [36, 37], CBV imaging also partly removes the effect of overestimates about BVF. This is therefore a suitable method for approximating the value of $V_0$. In addition, this study concentrated on explaining the influence of BVF on hemodynamic model estimation, and the results demonstrated the importance of taking advantage of actual BVF information in the estimation procedure. The argument about the origin of the two modalities were beyond the scope of this paper.

## Conclusion

The present study presented the first empiric attempt to derive the actual $V_0$ from data obtained using CBV imaging, with the aim of augmenting the existing estimation schemes. The results show that $V_0$ significantly influences the estimation results within a single-region model estimation and DCM. Using the actual $V_0$ can provide more reliable and accurate parameterizations and model predictions, and improve brain connectivity estimation based on fMRI data.

## Abbreviations

fMRI: functional magnetic resonance imaging
BOLD: blood oxygen level dependent
BVF: blood volume fraction
CBV: cerebral blood volume
DCM: dynamic causal modeling
ROI: region of interest
dHb: deoxyhemoglobin
DSC: dynamic susceptibility contrast
LPM: left primary motor
RPM: right primary motor
